# Stratifying diffuse large B-cell lymphoma patients treated with chemoimmunotherapy: GCB/non-GCB by immunohistochemistry is still a robust and feasible marker

**DOI:** 10.18632/oncotarget.7495

**Published:** 2016-02-19

**Authors:** Ana Batlle-López, Sonia González de Villambrosía, Mazorra Francisco, Sefora Malatxeberria, Anabel Sáez, Carlos Montalban, Lydia Sánchez, Juan F. Garcia, Eva González-Barca, Andrés López-Hernández, MC Ruiz-Marcellan, Manuela Mollejo, Carlos Grande, Kristy L. Richards, Eric D. Hsi, Alexandar Tzankov, Carlo Visco, Zijun Y. Xu-Monette, Xin Cao, Ken H. Young, Miguel Ángel Piris, Eulogio Conde, Santiago Montes-Moreno

**Affiliations:** ^1^ Departments of Haematology and Pathology, Hospital Marques de Valdecilla, and IDIVAL, Santander, Spain; ^2^ Biobanco del Sistema Sanitario Público de Andalucía, Granada, Spain; ^3^ MD Anderson Cancer Center, Madrid, Spain; ^4^ Biotechnology Programme, Histology and Immunohistochemistry Core Unit, Spanish National Cancer Research Centre (CNIO), Madrid, Spain; ^5^ Pathology, MD Anderson Cancer Center, Madrid, Spain; ^6^ Department of Haematology, Hospital de Bellvitge (ICOIRO), Barcelona, Spain; ^7^ Departments of Pathology and Haematology, Hospital Vall d'Hebron, Barcelona, Spain; ^8^ Hospital Virgen de la Salud, Toledo, Spain; ^9^ Hematology, Hospital 12 de Octubre, Madrid, Spain; ^10^ Department of Hematology-Oncology, University of North Carolina School of Medicine, Chapel Hill, NC, USA; ^11^ Department of Clinical Pathology, Cleveland Clinic, Cleveland, OH, USA; ^12^ University Hospital, Basel, Switzerland; ^13^ Department of Hematology, San Bortolo Hospital, Vicenza, Italy; ^14^ Department of Hematopathology, University of Texas MD Anderson Cancer Center, Houston, TX, USA

**Keywords:** MYC, BCL2, BCL6, non-GCB and GCB, DLBCL

## Abstract

Diffuse large B cell lymphoma (DLBCL) is a heterogeneous group of aggressive lymphomas that can be classified into three molecular subtypes by gene expression profiling (GEP): GCB, ABC and unclassified. Immunohistochemistry-based cell of origin (COO) classification, as a surrogate for GEP, using three available immunohistochemical algorithms was evaluated in TMA-arranged tissue samples from 297 patients with *de novo* DLBCL treated by chemoimmunotherapy (R-CHOP and R-CHOP-like regimens). Additionally, the prognostic impacts of *MYC*, *BCL2*, *IRF4* and *BCL6* abnormalities detected by FISH, the relationship between the immunohistochemical COO classification and the immunohistochemical expression of MYC, BCL2 and pSTAT3 proteins and clinical data were evaluated.

In our series, non-GCB DLBCL patients had significantly worse progression-free survival (PFS) and overall survival (OS), as calculated using the Choi, Visco-Young and Hans algorithms, indicating that any of these algorithms would be appropriate for identifying patients who require alternative therapies to R-CHOP. Whilst *MYC* abnormalities had no impact on clinical outcome in the non-GCB subtype, those patients with isolated *MYC* rearrangements and a GCB-DLBCL phenotype had worse PFS and therefore might benefit from novel treatment approaches.

## INTRODUCTION

Diffuse large B-cell lymphoma (DLBCL) is the most prevalent subtype of mature B-cell neoplasms worldwide, accounting for approximately 30% of all non-Hodgkin lymphomas. A combination of rituximab with an anthracycline-based chemotherapy regimen, such as R-CHOP, produces a favorable outcome for most patients. Nevertheless, one third of patients will still relapse, of whom a proportion will suffer from refractory disease [1].

Although the International Prognostic Index (IPI) has proved to be a very useful clinical tool, and is widely used to risk-stratify patients [2], it is exclusively based on clinical variables and therefore fails to capture the biological heterogeneity of DLBCL, and does not identify potential targeted therapies.

In recent years, biological studies have shown that DLBCL is not a single disease but a group of disorders with specific signaling programs [3], some of which have been shown to benefit from specific novel treatments. Specifically, gene expression profiling (GEP) has identified three DLBCL subtypes: the germinal center B-cell-like (GCB) and the activated B cell-like (ABC) DLBCL subtypes, and unclassified cases (∼10%). GCB patients tend to have better survival than the ABC and unclassified DLBCL patients when treated by chemotherapy, with or without rituximab [4, 5]. New treatments including ibrutinib [6] or immunomodulators such as lenalidomide have shown very promising results in patients with an ABC or non-GCB DLBCL subtype [7], as determined by GEP and immunohistochemistry in separate clinical trials. Although preferential activity with bortezomib for cases with the refractory ABC/non-GCB subtype was suggested early on [8], recently published data concerning frontline VR-CAP do not indicate a significant difference with R-CHOP in the non-GCB type DLBCL [9], and the differential efficacy remains controversial.

For this reason, identification of cases that will not respond to conventional approaches but might benefit from targeted therapy has become a matter of considerable interest.

Several studies have demonstrated that immunohistochemical (IHC) algorithms can reproduce GEP classification and potentially be used for the prognostic stratification of DLBCL [10–12]. Of these, the Hans, Visco-Young and Choi algorithms have shown the highest concordance with the cell of origin (COO) as defined by GEP. However, several clinical studies investigating the ability of COO IHC algorithms to prognostically stratify DLBCL patients treated with anthracycline-based chemotherapy with or without rituximab have yielded conflicting results [13–18].

Recurrent non-random chromosomal rearrangements leading to the deregulated expression of MYC, BCL6 and BCL2 in DLBCLs have been reported to have mixed associations with prognosis [17, 19–24]. Concurrent *MYC* and *BCL2* rearrangements [20, 25–27] are associated with poor outcome in DLBCL and are commonly found in GCB-type DLBCLs [28]. *IRF4* rearrangements are associated with a GCB phenotype in grade 3FL and DLBCL, and with younger age and favorable outcome [29]. Constitutive activation of STAT3 is preferentially detected in the non-GCB subtype of DLBCL, and seems to favor proliferation and survival of lymphoma cells [30]. However, the prognostic impact of this molecule is yet to be established.

In an attempt to confirm that IHC algorithms can be used to prognostically stratify DLBCL patients, we compared the Hans, Choi and Visco-Young algorithms in a cohort of 297 diagnostic samples from patients with R-CHOP-treated DLBCL. Also, to establish whether deregulation of specific oncogenes may further classify COO-based DLBCL subtypes, adding meaningful prognostic information, we performed FISH of *BCL6*, *BCL2*, *IRF4* and *MYC* and evaluated the expression of MYC, BCL2, BCL6 and pSTAT3 proteins in the same samples.

## RESULTS

**Figure 1:**
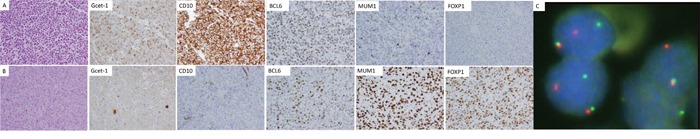
**A.** Immunohistochemistry was used to identify DLBCL subtypes. A prototypical example of GCB-type DLBCL is shown, as classified by the three algorithms considered. The case shows positive staining for GCET1, CD10, BCL6 and negative (or below the threshold) staining for MUM1 and FOXP1. **B.** A case of non-GCB/ABC subtype, classified by the three algorithms, is shown. The case has immunohistochemical expression of MUM1 and FOXP1 and negative (or below the threshold) staining for Gcet1, CD10 and BCL6. **C.** FISH using the LSI *MYC* break-apart probe on a tissue sample of a patient with a GCB DLBCL with rearrangement of the *MYC* gene.

The COO of 272 (91%), 282 (95%) and 275 (92%) of the 297 DLBCL tumor samples could be classified by the Hans, Visco-Young and Choi algorithms, respectively (Figure 1). No significant differences were observed between the IHC phenotypes with respect to age or extranodal involvement. However, the non-GCB group of patients had a higher IPI score (p≤0.011) and more frequently presented with stage III or IV disease (p≤0.001). Non-GCB cases had higher LDH levels and worse performance status than GCB cases as classified by the Hans and Visco-Young algorithms (Table 1 and Supplementary Tables 1 and 2).

**Table 1:** Clinical characteristics and differences in MYC, BCL2, BCL6, pSTAT3 and IRF4 changes according to cell of origin type

	Choi classification (n=275)					Visco-Young classification (n=282)					Hans classification (n=272)				
	GCB (n=120)		Non-GCB (n=155)			GCB (n=112)		Non-GCB (n=170)			GCB (n=115)		Non-GCB (n=157)		
**Cohort**	No	%	No	%	p	No	%	No	%	p	No	%	No	%	p
IPI score High (3-5)	43 of 116	37	77 of 146	53	**0.011**	39 of 107	36	83 of 159	52	**0.011**	38 of 112	34	84 of 151	56	**<0.001**
MYC															
r*MYC*	9 of 85	11	6 of 111	6	0.176	9 of 79	11.5	6 of 120	5	**0.095**	9 of 85	11	6 of 116	5	0.149
g*MYC*	20 of 81	25	17 of 102	17	0.179	21 of 70	28	16 of 109	15	**0.03**	21 of 80	26	16 of 108	15	0.051
BCL2															
r*BCL2*	30 of 79	38	5 of 104	5	<0.001	30 of 78	38	6 of 107	6	**0.000**	31 of 80	39	4 of 105	4	**<0.001**
g*BCL2*	5 of 77	7	20 of 101	20	**0.011**	5 of 76	7	20 of 104	19	**0.015**	5 of 77	7	20 of 103	19	**0.013**
BCL6															
*rBCL6*	11 of 72	15	38 of 92	41	**<0.001**	11 of 68	16	39 of 98	40	**0.001**	12 of 71	17	38 of 96	40	**0.002**
g*BCL6*	15 of 81	19	15 of 92	16	0.701	13 of 77	17	17 of 98	17	0.936	15 of 79	19	15 of 97	16	0.536
IRF4															
*rIRF4*	1 of 20	5	2 of 53	4	0.623	1 of 16	6	2 of 58	3	0.524	1 of 22	4.5	2 of 52	4	0.659
*gIRF4*	0 of 9	0	4 of 30	13	0.333	0 of 8	0	4 of 32	12.5	0.393	0 of 10	0	4 of 30	13	0.3
DHL M*YC/BCL* 2+ (n=4)	4 of 55	7	1 of 68	1.5	0.123	4 of 55	7	1 of 70	1	0.117	4 of 55	7	1 of 70	1	0.117

Abbreviations: GCB: germinal center B-cell-like; DLBCL, diffuse large B-cell lymphoma; r*MYC*: rearrangement of *MYC*; *gMYC*: gain of the *MYC* locus; r*BCL2*: rearrangement of *BCL2*; *gBCL2*: gain of *BCL2* locus; *rBCL6* rearrangement of *BCL6*; *gBCL6*: gain of *BCL6* locus. IPI score ranges from 0 to 5, with 0 indicating the absence of prognostic factors and 5 indicating the presence of all prognostic factors. The IPI score was stratified by the proposed RIPI score. pSTAT3, phosphorylated STAT3 protein expression.

Comparing GEP with the results of the Hans, Choi and Visco-Young algorithms, revealed discrepancies in the COO classification in only 14%, 13% and 8% patients, respectively [12].

### MYC, BCL2, BCL6 and pSTAT3 protein expression and correlation with COO phenotypes

45% of the evaluable samples (62/140) showed MYC overexpression. 72% and 64% of patients with MYC protein overexpression also had high LDH levels (p=0.011) and high IPI scores (p=0.029), respectively (Supplementary Table 3). MYC overexpression was not associated with a particular COO phenotype (Supplementary Table 4). MYC and BCL2 double-positivity was found in 34% of evaluable cases (42/123 cases). As previously described, MYC and BCL2 double-positive (DP) cases determined by IHC were commonly found to have a non-GCB phenotype (p=0.007, p=0.018 and p=0.002 for the Visco-Young, Hans and Choi algorithms, respectively) (Supplementary Table 4). Most patients coexpressing the MYC and BCL2 proteins were aged over 60 years (74%), had stage III or IV disease (74%), and 36% of cases had two or more extranodal sites involved and were therefore associated with high-risk IPI (p=0.003; Supplementary Table 5). 15% and 24% of MYC and BCL2 double-positive cases had *MYC* and *BCL2* rearrangements, respectively (p=0.030 and p=0.938, Supplementary Table 6).

Isolated BCL2 overexpression was not associated with the COO classification, and there were no associations between BCL2 protein expression and any of the clinical or biological features analyzed in our series (data not shown).

pSTAT3 protein expression was detected in 34% (47/139) of evaluable cases and was associated with advanced-stage disease (p=0.003) and high IPI score (p=0.045; Supplementary Table 5). Its expression was more frequent in cases with elevated MYC expression (p=0.006) (Supplementary Table 3).

### *MYC, BCL6, BCL2* and *IRF4* abnormalities

*MYC* rearrangements were observed in 7% of patients (15 out of 206 cases) (Table 2 and Figure 1), 30% of which had additional cytogenetic abnormalities, *BCL2* rearrangements and *MYC* gains being the most common added gene aberrancies (in 4 out of 13, and 2 out of 15 evaluable cases, respectively). *MYC* rearrangements were more frequent in patients younger than 60 years of age (p=0.029), with more than two extranodal localizations (p=0.009), and in the high-risk IPI group (p=0.051). We found no difference in the distribution of *MYC* rearrangements with respect to the COO phenotype. No significant correlation was observed between the presence of *MYC* rearrangements and MYC protein expression (5 out of 63 cases with MYC protein overexpression had *MYC* rearrangements).

**Table 2:** Clinical features with respect to the presence of *MYC*, *BCL2* and *BCL6* rearrangements

Patients, No. (%)	MYC (n=15/206) Rearranged		BCL2 (n=36/187) Rearranged		BCL6 (n=51/171) Rearranged	
		P		P		P
Age, years						
> 60	5 (36)	**0.029**	20 (62)	0.920	32 (65)	0.581
≤ 60	9 (64)		12 (38)		17 (35)	
Ann Arbor stage						
I-II	3 (21)	0.153	14 (44)	0.381	18 (37)	0.735
III-IV	11 (78)		18 (56)		30 (62)	
LDH						
Low	3 (23)	0.097	10 (38)	0.835	12 (30)	**0.021**
> upper limit of normal	10 (77)		16 (62)		28 (70)	
Extranodal sites						
≤ 1	5(35)	**0.009**	20 (74)	0.656	28 (72)	0.695
≥ 2	9(64)		7 (26)		11 (28)	
ECOG †						
PS ≤ 1	11(79)	0.473	8 (31)	0.780	25 (64)	0.659
PS > 1	3 (21)		8 (31)		14 (36)	
IPI score ‡						
Low (0-2)	4 (29)	**0.051**	19 (58)	0.268	24 (50)	0.533
High (3-5)	10 (71)		14 (42)		24 (50)	

Abbreviations: IPI, International Prognostic Index; LDH, lactate dehydrogenase; †ECOG PS, ranging from 0 to 4, where a higher score indicates greater impairment. ‡ IPI score, ranging from 0 to 5, where 0 indicates the absence of prognostic factors, and 5 indicates the presence of all prognostic factors. The IPI score was stratified by the proposed RIPI score.

*MYC* gains (Supplementary Table 7) were observed in 19% of patients and were more frequently observed in the group of patients with normal LDH values (p=0.032). Cases with *MYC* gains expressed MYC, BCL2 and BCL6 protein in 31%, 56% and 84% of cases, respectively. *MYC* gains were associated with gains in *BCL2* (p=0.002) and *BCL6* (p=0.002), but there was no association with other cytogenetic, biological or clinical features.

*BCL2* rearrangements were detected in 19% of patients (Table 2). There was no association between the presence of any type of *BCL2* gene abnormality and the main clinical features studied. *BCL2* rearrangements were almost entirely restricted to the GCB phenotype (Table 1, p<0.001). There was a close correlation between the presence of a *BCL2* rearrangement and BCL2 protein overexpression (p=0.003; 95% of cases with the *BCL2* rearrangement expressed the BCL2 protein; data not shown). *BCL2* gains were observed in 14% of patients and were more frequently detected in the non-GCB phenotype group (p=0.013; 80% of cases with *BCL2* amplification had a non-GCB phenotype; Table 1).

*BCL6* rearrangements were observed in 30% (51 out of 171) of DLBCL patients, were associated with a non-GCB phenotype (Table 1, p<0.001; 70% of cases) [38] and were more frequent in the group of patients with elevated LDH (p=0.021) (Table 2). However, there was no association with other clinical features. *BCL6* gains, observed in 17% of patients, showed no significant association with any of the clinical or phenotypic features analyzed. In contrast to the *BCL6* rearrangements, there was a direct relation between the presence of *BCL6* gains and BCL6 protein expression (Table 2; p=0.02).

Double rearrangements were detected in 10 of 178 cases: there were concurrent *BCL2/MYC* rearrangements in 4 cases, *BCL2/BCL6* rearrangements in 5 cases and *MYC/BCL6* in 1 case. Taken together, 80% of double-rearrangement DLBCL cases had a GCB phenotype (p=0.029), and there was a higher absolute proportion of them in patients aged over 60 years (p=0.073) and with elevated LDH values (p=0.053), although these trends were not statistically significant (Supplementary Table 8). 37% of patients presented involvement of two or more extranodal sites, and 60% had stage III or IV disease. Considering only double-hit cases (*BCL2/MYC* and *BCL6/MYC* patients), no significant association with any specific clinical or biological feature was detected, probably due to the small number of cases available.

*IRF4* rearrangements and gains were observed in 3 out of 76, and 4 out 41 assessable patients, respectively. 67% and 100% of *IRF4* rearrangement and gain cases, respectively, had a non-GCB phenotype (Table 1). There was no association between the *IRF4* rearrangement and the clinical variables. An association between *IRF4* and *BCL2* gains was observed (p=0.035). In contrast to previously published data [29], in our series, 60% of patients with *IRF4* rearrangements were over 60 years old. It should be noted, however, that 60% of the patients in this study were older than 60 years of age.

### Survival analysis

All clinical and biological variables were evaluated for their ability to predict response and outcome. In the univariate analysis, stage III-IV disease was associated with worse OS (p=0.016) and PFS (p<0.001), as were elevated LDH levels, age ≥60 years, poorer performance status, extranodal involvement and high IPI score. As in previous studies, the independent prognostic impact of all IPI variables was maintained in the multivariate analysis.

**Figure 2:**
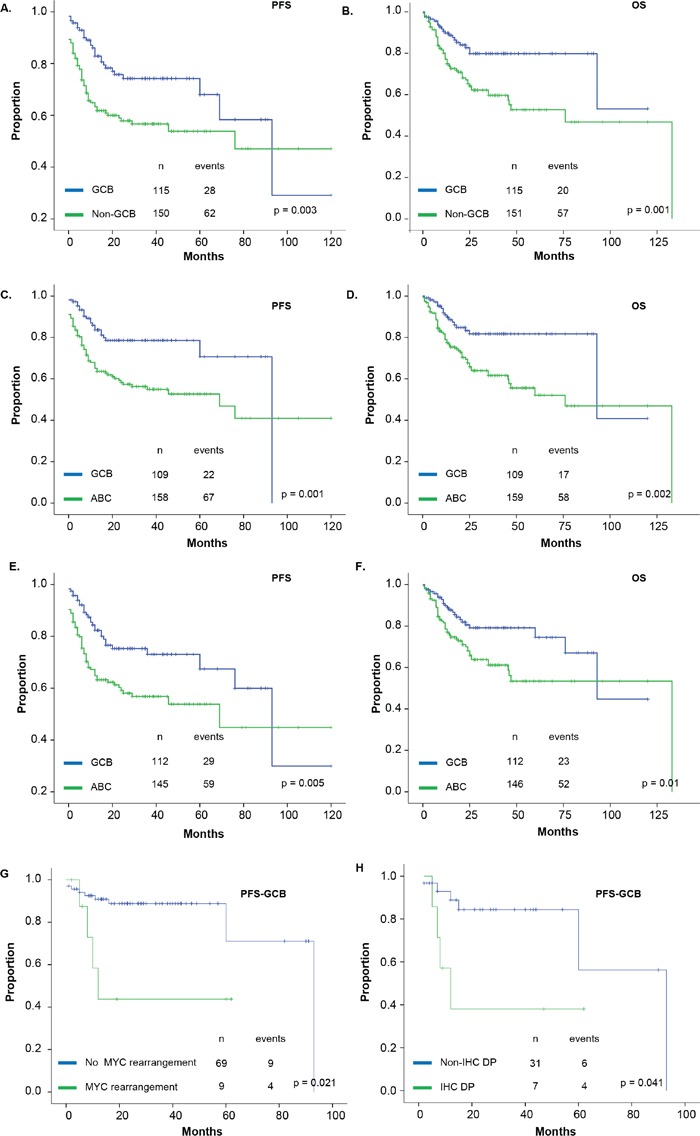
Univariate prognostic analysis of progression-free survival (PFS) and overall survival (OS) PFS and OS of GCB and non-GCB DLBCL using the Hans (A and B), Visco-Young (C and D), and Choi (E and F) algorithms. (G-I) PFS of *MYC* rearrangements in GCB-DLBCL patients using the Visco-Young, Han and Choi algorithms, respectively. Abbreviations: IPI, International Prognostic Index; ‡ The IPI score ranges from 0 to 5, with 0 indicating the absence of prognostic factors and 5 indicating the presence of all prognostic factors. The IPI score was stratified by the proposed RIPI score. IHC DP: Immunohistochemical double-positive.

Considering the Visco-Young, Choi and Hans algorithms, the non-GCB subgroup was associated with significantly worse PFS (p=0.001, p=0.005 and p=0.003, respectively) and OS (p=0.002, p=0.001 and p=0.001, respectively) (Table 3 and Figure 2A and 2B). In the multivariate analysis (Table 4), COO classification remained an independent prognostic factor of PFS and OS for all the algorithms (Table 4, hazard ratio [HR] 1.7-2.1; p<0.03). The algorithms showed close agreement with each other, with a level of concordance of 88.6-90.3 % (multi-rater kappa, k=0.8).

**Table 3:** Progression-free survival (PFS) and overall survival (OS) with respect to the IPI, cell of origin, immunohistochemical double-positive cases and gene abnormalities of the *MYC*, *BCL2* and *BCL6* loci

	PFS		OS	
	RR (95% CI)	p	RR (95% CI)	p
Age, years > 60 ≤ 60	13 (0.8-2.1)	0.198	2.2 (1.3-3.7)	**0.003**
Ann Arbor stage I-II III-IV	2.4 (1.5-3.9)	**<0.001**	1.8 (1.1-2.9)	**0.019**
LDH Low > upper limit of normal	2.5 (1.5-4.1)	**<0.001**	2.4 (1.4-4.2)	**0.002**
Extranodal sites ≤ 1 ≥ 2	2.1 (1.3-3.2)	**0.001**	2.0 (1.3-3.3)	**0.003**
ECOG † PS ≤ 1 PS > 1	2.3 (1.5-3.6)	**<0.001**	2.6 (1.6-4.2)	**<0.001**
IPI score ‡ Low (0-2) High (3-5)	2.5 (1.6-3.9)	**<0.001**	2.7 (1.7-4.4)	**<0.001**
Cell of origin subtype Hans Classification Non-GCB GCB	1.9 (1.2-3.0)	**0.003**	2.3 (1.3-3.8)	**0.001**
Choi Classification Non-GCB GBC	1.8 (1.2-2.9)	**0.005**	1.9 (1.1-3.1)	**0.010**
Visco-Young Classification Non-GCB GCB	2.3 (1.4-3.7)	**0.001**	2.3 (1.3-4.0)	**0.002**
MYC r*MYC* g*MYC* MYC protein expression	1.5 (0.6-3.7) 0.5 (0.2-1.2) 0.9 (0.5-1.6)	0.279 0.173 0.803	1.1 (0.4-3.2) 0.6 (0.3-1.4) 0.9 (0.5-1.8)	0.738 0.273 0.979
BCL2 r*BCL2* gBCL2 BCL2 protein expression	0.7 (0.4-1.6) 0.8 (0.3-1.8) 1.2 (0.7-2.2)	0.477 0.648 0.410	0.3 (0.1-1.0) 1.1 (0.5-2.5) 1.0 (0.5-2.0)	0.067 0.687 0.918
BCL6 r*BCL6* g*BCL6* BCL6 protein expression	1.0 (0.6-1.9) 0.7 (0.3-1.5) 0.6 (0.4-1.0)	0.784 0.384 0.077	1.2 (0.6-2.1) 0.8 (0.4-1.6) 0.6 (0.4-1.0)	0.514 0.565 **0.048**
pSTAT3 protein expression	0.9 (0.5-1.5)	0.739	0.8 (0.4-1.5)	0.484
IRF4 r *IRF4* g *IRF4*	- 0.9 (0.1-7.4)	0.450 0.981	- 0.4 (0.1-3.4)	0.500 0.440
Double-HIT (FISH) c-*MYC+/BCL2*+(IHC)	0.8 (0.2-4.6) 1.1 (0.6-2.0)	0.874 0.625	0.6 (0.1-4.7) 0.9 (0.4-1.8)	0.646 0.813

Abbreviations: r*MYC*: rearrangement of *MYC*; g*MYC*: gain of *MYC* locus; r*BCL2*: rearrangement of *BCL2*; g*BCL2*: gain of *BCL2* locus; r*BCL6* rearrangement of *BCL6*; g*BCL6*: gain of *BCL6* locus; DLBCL, diffuse large B-cell lymphoma; ECOG PS, Eastern Cooperative Oncology Group performance status; GCB, germinal center B-cell-like; GEP, gene expression profiling; IPI, International Prognostic Index; DHL, double-hit lymphoma; IHC, immunohistochemistry. ‡ The IPI score ranges from 0 to 5, with 0 indicating the absence of prognostic factors, and 5 indicating the presence of all prognostic factors. The IPI score was stratified by the proposed RIPI score.

**Table 4:** Multivariate prognostic analysis of progression-free survival (PFS) and overall survival (OS) according to COO classification algorithms

	PFS		OS	
	RR (95% CI)	p	RR (95% CI)	p
IPI score ^‡^	2.3 (1.5-3.6)	**<0.001**	2.49 (1.5-4.0)	**<0.001**
Choi classification	1.7 (1.1-2.7)	**0.016**	1.7 (1.1-2.8)	**0.027**
IPI score ^‡^	2.2 (1.4-3.4)	**<0.001**	2.3 (1.4-3.7)	**0.001**
Hans classification	1.7 (1.1-2.8)	**0.015**	2.0 (1.2-3.4)	**0.007**
IPI score ^‡^	2.2 (1.4-3.4)	**<0.001**	2.4 (1.5-4.0)	**<0.0001**
Visco-Young classification	2.1 (1.3-3.5)	**0.003**	2.1(1.2-3.6)	**0.006**

Abbreviations: IPI, International Prognostic Index;

‡ The IPI score ranges from 0 to 5, with 0 indicating the absence of prognostic factors, and 5 indicating the presence of all prognostic factors. The IPI score was stratified by the proposed RIPI score.

As seen in a previous study [39], patients with *MYC* rearrangements and GCB phenotype had a significantly shorter PFS (p=0.035, p=0.004 and p=0.021, for the Hans, Choi and Visco-Young algorithms, respectively) (Figure 2G, Supplementary Table 9). None of the algorithms was of prognostic value with respect to OS, although with the Visco-Young algorithm, patients with a GCB phenotype and *MYC* rearrangements showed a non-significant tendency towards a lower OS (p=0.098), (Supplementary Table 10). Neither *BCL2* nor *BCL6* abnormalities had a clear impact on outcome, either in the series as a whole or in the specific COO subtypes. In contrast, in the entire series, BCL6 expression tended to be associated with better PFS (p=0.077) and OS (p=0.048).

In our series, patients coexpressing MYC and BCL2 did not show worse PFS or OS. The presence of MYC, BCL2 and/or pSTAT3-positive protein expression was not associated with poorer PFS or OS. This might be due to the limited number of cases with available IHC data for these markers. Unavailability was most commonly due to tissue exhaustion.

## DISCUSSION

Prognostic stratification of DLBCL patients has customarily been based on the clinical parameters comprising the International Prognostic Index (IPI). This classification continues to be the standard clinical tool for predicting the outcome of DLBCL patients in the rituximab era [40]. However, it does not capture the biological heterogeneity of the disease and does not identify potential targets for therapy. GEP studies have identified three subtypes of DLBCL with distinct biological signatures: the GCB, the ABC and the unclassified groups. Although gene expression in formalin-fixed paraffin-embedded tissue [41] is probably the preferred method for differentiating these two DLBCL subtypes, it is not yet available in most centers. Classification using digital PCR assay has shown promising results [41, 42]. Nevertheless, a subgroup of ∼10% of cases would remain unclassified even after using these methods.

Several studies have shown that IHC algorithms can discriminate GCB and non-GCB DLBCL subtypes with a high concordance with GEP, but there is still a reluctance to using them in the clinical setting. In this study, the Hans, Visco-Young and Choi algorithms were used as surrogates for GEP-based COO molecular classification. Variation due to the technique was reduced by centralized staining and scoring. All the IHC approaches used were able to distinguish the two prognostic subgroups, and showed substantial agreement among themselves, and a similar correlation with GEP [12]. The results were comparable to those we and other researchers had obtained in previous studies [12, 43]. In agreement with previous reports [10, 44, 45], the non-GCB subgroup had a worse outcome, with a shorter PFS and OS, and this prognostic impact was maintained in the multivariate analysis. These results indicate that non-GCB patients require novel treatment approaches, including the addition of novel compounds, in order for the responses to be sustained. Indeed, a number of different new drugs including lenalidomide, some proteasome inhibitors, and the BTK inhibitor ibrutinib (PCI-32765) have recently shown promising results, especially in non-GCB/ABC DLBCL patients [7, 46–49]. Whether IHC and GEP are equally capable of distinguishing patients who could benefit from these novel compounds requires further investigation since the results published so far emerged from different methods of determination.

To refine the prognostic stratification of patients after GCB/non-GCB IHC classification, we analyzed the effect of *MYC*, *BCL2*, *BCL6* and *IRF4* abnormalities as detected by FISH. We detected *MYC* rearrangements in 7% of the cases, only 30% of which had additional cytogenetic abnormalities. The negative impact on prognosis for the isolated *MYC* rearrangement, as a single cytogenetic abnormality, was restricted to patients with a GCB phenotype and was associated with a shorter PFS. Thus, although the prognostic implication of the finding was not confirmed in the multivariate analysis, probably because of the small number of patients with this abnormality, our results [39] confirm that the favorable prognosis of GCB-type DLBCL is overcome by the negative effect of *MYC* rearrangements. For this reason, a systematic investigation of *MYC* rearrangements, at least within the GCB subgroup of patients, would be worthwhile. Despite their lower PFS, these patients did not have a significantly worse OS, indicating that these particular cases may be more aggressive but can be rescued with savage therapies. Together, these findings suggest that GCB-type DLBCL patients carrying a *MYC* rearrangement would probably benefit from treatment strategies other than R-CHOP, for example, more aggressive chemotherapy schemes such as R-DAEPOCH, or the addition of MYC inhibitors. Indeed, several small molecules have been identified as MYC inhibitors, although none is currently in clinical use [50]. In a recently employed approach, MYC expression is blocked by impairing a BET bromodomain protein, a strategy that has proved effective both *in vitro* and in xenograft models in a range of hematological neoplasms, including Burkitt lymphoma [51–53]. It should be noted that *MYC* rearrangements and MYC expression were not correlated in this study, indicating that MYC expression might not be the best screening method for detecting MYC rearrangements in cases of DLBCL NOS. Almost half of the patients expressing MYC protein also had a high level of pSTAT3 expression. pSTAT3 was preferentially, but not exclusively, overexpressed in the non-GCB type DLBCL cases [30, 34], but, in contrast to other groups [54, 55], was not associated with an unfavorable outcome. Thus, our findings are consistent with those of previous studies demonstrating that pSTAT3 signaling is involved in regulating MYC expression [56], so pSTAT3 inhibitors could also be used in those cases [57, 58].

As in previous studies, double-hit DLBCLs seem to be restricted to GCB-type DLBCLs. However, the small number (5/100) and the short follow-up of these cases prevented us from determining the prognostic impact in terms of OS and PFS. Most patients with concurrent MYC and BCL2 overexpression had high-risk IPI features and, consistent with previous findings, this phenotype was found more frequently than the double-hit with FISH and, unlike with true double-hits, was associated with a non-GCB phenotype [33, 59, 60]. Notably, only 15% and 24% of double-IHC cases had *MYC* and *BCL2* rearrangements, respectively, suggesting that there must be other mechanisms that induce MYC and BCL2 overexpression.

In our cohort, *BCL2* rearrangements, which were observed in 19% of the patients, were also mainly restricted to the GCB phenotype [22]. However, in contrast to the results from previously published series [22, 61], we found no clear negative effect on those patients' outcome, possibly due to the small number of cases featuring the abnormality. Also, the adverse prognostic implication of *BCL2* rearrangements might apply only to younger patients, so the different selection criteria used in the studies might explain the observed differences [62]. Nevertheless, BCL2 inhibition is an attractive target for therapy, as illustrated by the fact that at least three BCL2 antagonists are currently undergoing clinical trials for cancer treatment [63].

In accordance with previous findings, *BCL6* rearrangements that were usually detected in the non-GCB patient group [38] were not clearly associated with BCL6 protein overexpression, and their detection was of no independent prognostic consequence with respect to OS or PFS. The prognostic contribution of BCL6 protein expression remains controversial [64–66] and, as with some previous studies, we have shown that BCL6 expression is associated with a better OS in the rituximab era. Since BCL6 expression is almost completely restricted to germinal center cells and some lymphomas, BCL6 is an attractive target for therapy with several specific BCL6 inhibitors currently being investigated [67].

In summary, our results confirm that COO classification based on IHC is a reproducible and practical approach to stratifying DLBCL patients into GCB and non-GCB phenotypes, the latter being associated with a higher risk of progression and reduced survival when conventional R-CHOP regimens are used. These patients might benefit from treatment with novel compounds that target signaling pathways known to be constitutively activated in this particular lymphoma subtype.

Identification of *MYC* rearrangements enables GCB-DLBCLs to be stratified into prognostic groups. GCB patients with isolated *MYC* rearrangements have a higher risk of progression and might benefit from adding new therapeutic approaches, including drugs that inhibit MYC function. Despite the lack of prognostic information, identification of certain proteins, such as BCL6 or pSTAT3, might be of interest as potential druggable target molecules.

## PATIENTS AND METHODS

### Patient selection

The study population consisted of a retrospective series of 297 unselected *de novo* cases of DLBCL obtained from various centers in Spain (153 cases), and other institutions involved in the International DLBCL Rituximab-CHOP Consortium Program Study in the USA (144 cases). Some of these cases have already been published [31, 32]. The study was reviewed and approved by each of the participating Institutional Review Boards. The overall collaborative study and the study protocol and sampling methods were approved by the Institutional Review Board of the Hospital Universitario Marqués de Valdecilla/IDIVAL, Spain. Histopathological criteria used for their diagnosis and classification were those of the WHO Classification [3]. Cases associated with HIV or HCV infections or previous immunosuppressive treatments were excluded, as were cases diagnosed as T-cell histiocyte-rich B-cell lymphoma, primary mediastinal B-cell lymphoma cases, cutaneous LBCL, intravascular LBCL, EBV+DLBCL and those histologically associated with a follicular lymphoma component. All patients were treated as part of their routine care with standard treatment protocols using a combination of anthracycline-based regimens (6-8 cycles in most cases) and rituximab. Follow-up data on progression-free survival (PFS) and overall survival (OS) were available for 279 (94%) and 283 (95%) patients, respectively.

### Immunohistochemistry, TMA construction and FISH analysis

Histopathological evaluation, immunohistochemistry and FISH analysis were performed in a central laboratory by experienced hematopathologists and cytogeneticists. All DLBCL cases were histologically confirmed and representative areas were selected. Detailed IHC staining procedures are described in the Supplementary Data. The cut-offs for the IHC markers were BCL2 >70%, MYC >40% and pSTAT3 >10%, based on published data [33, 34]. Scoring of GCET1, MUM1, CD10, BCL6, and FOXP1 was based on the cut-offs used by Choi et al [11], Hans et al [10] and Visco Young [35]. Immunoreactivity was scored by two independent pathologists (SMM and SMU) and the percentage of tumor-cell staining was estimated by visual inspection and categorized into the appropriate decile. Disagreements were resolved by joint review using a multiheaded microscope. For each case the most representative core, with the highest percentage of neoplastic cells, was selected.

FISH analyses were performed on 3-μm TMA tissue sections following standard procedures [36, 37] (see Supplementary Data for details). At least 100 intact, non-overlapping nuclei were analyzed from each TMA core. Discordant duplicates were reevaluated by two observers (AB, SG). Nuclei were scored as rearranged if at least one split orange-green signal was observed. Gains were reported when three or more fusion signals were observed. Control values were previously established on the basis of samples of 10 controls (mean plus or minus three standard deviations). The cut-off values for chromosome gain or rearrangement were 15% in each case.

### Statistical analysis

Patient characteristics and response rates were compared using the X^2^ or Fisher's exact test, depending on the number of observations. PFS was defined as the time from initial diagnosis to progressive disease under therapy, failure to achieve complete remission, additional therapy, relapse, or death from any cause. OS was defined as the time from initial diagnosis to death. PFS and OS were analyzed using the log-rank test and illustrated as Kaplan-Meier plots. A Cox proportional hazards multivariate regression model was derived. Differences between the results of comparative tests were considered significant for two-sided values of p<0.05. The multi-rater kappa was used to assess the concordance between the algorithms.

## SUPPLEMENTARY TABLES



## References

[1] Sehn LH (2012). Paramount prognostic factors that guide therapeutic strategies in diffuse large B-cell lymphoma. Hematology.

[2] Ziepert M, Hasenclever D, Kuhnt E, Glass B, Schmitz N, Pfreundschuh M, Loeffler M (2010). Standard International prognostic index remains a valid predictor of outcome for patients with aggressive CD20+ B-cell lymphoma in the rituximab era. Journal of clinical oncology.

[3] Swerdlow SH, Campo E, Harris NL, Jaffe ES, Pileri SA, Stein H, Thiele J, JW V (2008). WHO Classification of Tumors of Haematopoietic and Lymphoid Tissues.

[4] Alizadeh AA, Eisen MB, Davis RE, Ma C, Lossos IS, Rosenwald A, Boldrick JC, Sabet H, Tran T, Yu X, Powell JI, Yang L, Marti GE, Moore T, Hudson J, Lu L (2000). Distinct types of diffuse large B-cell lymphoma identified by gene expression profiling. Nature.

[5] Rosenwald A, Wright G, Chan WC, Connors JM, Campo E, Fisher RI, Gascoyne RD, Muller-Hermelink HK, Smeland EB, Giltnane JM, Hurt EM, Zhao H, Averett L, Yang L, Wilson WH, Jaffe ES (2002). The use of molecular profiling to predict survival after chemotherapy for diffuse large-B-cell lymphoma. N Engl J Med.

[6] Wilson WH, Young RM, Schmitz R, Yang Y, Pittaluga S, Wright G, Lih CJ, Williams PM, Shaffer AL, Gerecitano J, de Vos S, Goy A, Kenkre VP, Barr PM, Blum KA, Shustov A (2015). Targeting B cell receptor signaling with ibrutinib in diffuse large B cell lymphoma. Nat Med.

[7] Nowakowski GS, LaPlant B, Macon WR, Reeder CB, Foran JM, Nelson GD, Thompson CA, Rivera CE, Inwards DJ, Micallef IN, Johnston PB, Porrata LF, Ansell SM, Gascoyne RD, Habermann TM, Witzig TE (2015). Lenalidomide combined with R-CHOP overcomes negative prognostic impact of non-germinal center B-cell phenotype in newly diagnosed diffuse large B-Cell lymphoma: a phase II study. Journal of clinical oncology.

[8] Dunleavy K, Pittaluga S, Czuczman MS, Dave SS, Wright G, Grant N, Shovlin M, Jaffe ES, Janik JE, Staudt LM, Wilson WH (2009). Differential efficacy of bortezomib plus chemotherapy within molecular subtypes of diffuse large B-cell lymphoma. Blood.

[9] Offner F, Samoilova O, Osmanov E, Eom HS, Topp MS, Raposo J, Pavlov V, Ricci D, Chaturvedi S, Zhu E, van de Velde H, Enny C, Rizo A, Ferhanoglu B (2015). Frontline rituximab, cyclophosphamide, doxorubicin, and prednisone with bortezomib (VR-CAP) or vincristine (R-CHOP) for non-GCB DLBCL. Blood.

[10] Hans CP, Weisenburger DD, Greiner TC, Gascoyne RD, Delabie J, Ott G, Muller-Hermelink HK, Campo E, Braziel RM, Jaffe ES, Pan Z, Farinha P, Smith LM, Falini B, Banham AH, Rosenwald A (2004). Confirmation of the molecular classification of diffuse large B-cell lymphoma by immunohistochemistry using a tissue microarray. Blood.

[11] Choi WW, Weisenburger DD, Greiner TC, Piris MA, Banham AH, Delabie J, Braziel RM, Geng H, Iqbal J, Lenz G, Vose JM, Hans CP, Fu K, Smith LM, Li M, Liu Z (2009). A new immunostain algorithm classifies diffuse large B-cell lymphoma into molecular subtypes with high accuracy. Clinical cancer research.

[12] Visco C, Li Y, Xu-Monette ZY, Miranda RN, Green TM, Tzankov A, Wen W, Liu WM, Kahl BS, d'Amore ES, Montes-Moreno S, Dybkaer K, Chiu A, Tam W, Orazi A, Zu Y (2012). Comprehensive gene expression profiling and immunohistochemical studies support application of immunophenotypic algorithm for molecular subtype classification in diffuse large B-cell lymphoma: a report from the International DLBCL Rituximab-CHOP Consortium Program Study. Leukemia.

[13] Zinzani PL, Dirnhofer S, Sabattini E, Alinari L, Piccaluga PP, Stefoni V, Tani M, Musuraca G, Marchi E, Falini B, Baccarani M, Pileri SA (2005). Identification of outcome predictors in diffuse large B-cell lymphoma. Immunohistochemical profiling of homogeneously treated de novo tumors with nodal presentation on tissue micro-arrays. Haematologica.

[14] Chang CC, McClintock S, Cleveland RP, Trzpuc T, Vesole DH, Logan B, Kajdacsy-Balla A, Perkins SL (2004). Immunohistochemical expression patterns of germinal center and activation B-cell markers correlate with prognosis in diffuse large B-cell lymphoma. The American journal of surgical pathology.

[15] van Imhoff GW, Boerma EJ, van der Holt B, Schuuring E, Verdonck LF, Kluin-Nelemans HC, Kluin PM (2006). Prognostic impact of germinal center-associated proteins and chromosomal breakpoints in poor-risk diffuse large B-cell lymphoma. Journal of clinical oncology.

[16] Moskowitz CH, Zelenetz AD, Kewalramani T, Hamlin P, Lessac-Chenen S, Houldsworth J, Olshen A, Chaganti R, Nimer S, Teruya-Feldstein J (2005). Cell of origin, germinal center versus nongerminal center, determined by immunohistochemistry on tissue microarray, does not correlate with outcome in patients with relapsed and refractory DLBCL. Blood.

[17] Copie-Bergman C, Gaulard P, Leroy K, Briere J, Baia M, Jais JP, Salles GA, Berger F, Haioun C, Tilly H, Emile JF, Banham AH, Mounier N, Gisselbrecht C, Feugier P, Coiffier B Immuno-fluorescence in situ hybridization index predicts survival in patients with diffuse large B-cell lymphoma treated with R-CHOP: a GELA study. Journal of clinical oncology.

[18] Gutierrez-Garcia G, Cardesa-Salzmann T, Climent F, Gonzalez-Barca E, Mercadal S, Mate JL, Sancho JM, Arenillas L, Serrano S, Escoda L, Martinez S, Valera A, Martinez A, Jares P, Pinyol M, Garcia-Herrera A (2011). Gene-expression profiling and not immunophenotypic algorithms predicts prognosis in patients with diffuse large B-cell lymphoma treated with immunochemotherapy. Blood.

[19] Akyurek N, Uner A, Benekli M, Barista I (2012). Prognostic significance of MYC, BCL2, and BCL6 rearrangements in patients with diffuse large B-cell lymphoma treated with cyclophosphamide, doxorubicin, vincristine, and prednisone plus rituximab. Cancer.

[20] Barrans S, Crouch S, Smith A, Turner K, Owen R, Patmore R, Roman E, Jack A (2010). Rearrangement of MYC is associated with poor prognosis in patients with diffuse large B-cell lymphoma treated in the era of rituximab. Journal of clinical oncology.

[21] Offit K, Lo Coco F, Louie DC, Parsa NZ, Leung D, Portlock C, Ye BH, Lista F, Filippa DA, Rosenbaum A (1994). Rearrangement of the bcl-6 gene as a prognostic marker in diffuse large-cell lymphoma. The New England journal of medicine.

[22] Barrans SL, Evans PA, O'Connor SJ, Kendall SJ, Owen RG, Haynes AP, Morgan GJ, Jack AS (2003). The t(14;18) is associated with germinal center-derived diffuse large B-cell lymphoma and is a strong predictor of outcome. Clinical cancer research.

[23] Horn H, Ziepert M, Becher C, Barth TF, Bernd HW, Feller AC, Klapper W, Hummel M, Stein H, Hansmann ML, Schmelter C, Moller P, Cogliatti S, Pfreundschuh M, Schmitz N, Trumper L (2013). MYC status in concert with BCL2 and BCL6 expression predicts outcome in diffuse large B-cell lymphoma. Blood.

[24] Savage KJ, Johnson NA, Ben-Neriah S, Connors JM, Sehn LH, Farinha P, Horsman DE, Gascoyne RD (2009). MYC gene rearrangements are associated with a poor prognosis in diffuse large B-cell lymphoma patients treated with R-CHOP chemotherapy. Blood.

[25] Johnson NA, Savage KJ, Ludkovski O, Ben-Neriah S, Woods R, Steidl C, Dyer MJ, Siebert R, Kuruvilla J, Klasa R, Connors JM, Gascoyne RD, Horsman DE (2009). Lymphomas with concurrent BCL2 and MYC translocations: the critical factors associated with survival. Blood.

[26] Li S, Lin P, Fayad LE, Lennon PA, Miranda RN, Yin CC, Lin E, Medeiros LJ (2012). B-cell lymphomas with MYC/8q24 rearrangements and IGH@BCL2/t(14;18)(q32;q21): an aggressive disease with heterogeneous histology, germinal center B-cell immunophenotype and poor outcome. Mod Pathol.

[27] Snuderl M, Kolman OK, Chen YB, Hsu JJ, Ackerman AM, Dal Cin P, Ferry JA, Harris NL, Hasserjian RP, Zukerberg LR, Abramson JS, Hochberg EP, Lee H, Lee AI, Toomey CE, Sohani AR (2010). B-cell lymphomas with concurrent IGH-BCL2 and MYC rearrangements are aggressive neoplasms with clinical and pathologic features distinct from Burkitt lymphoma and diffuse large B-cell lymphoma. Am J Surg Pathol.

[28] Caponetti GC, Dave BJ, Perry AM, Smith LM, Jain S, Meyer PN, Bast M, Bierman PJ, Bociek RG, Vose JM, Armitage JO, Aoun P, Fu K, Greiner TC, Chan WC, Sanger WG (2015). Isolated MYC cytogenetic abnormalities in diffuse large B-cell lymphoma do not predict an adverse clinical outcome. Leukemia & lymphoma.

[29] Salaverria I, Philipp C, Oschlies I, Kohler CW, Kreuz M, Szczepanowski M, Burkhardt B, Trautmann H, Gesk S, Andrusiewicz M, Berger H, Fey M, Harder L, Hasenclever D, Hummel M, Loeffler M (2011). Translocations activating IRF4 identify a subtype of germinal center-derived B-cell lymphoma affecting predominantly children and young adults. Blood.

[30] Ding BB, Yu JJ, Yu RY, Mendez LM, Shaknovich R, Zhang Y, Cattoretti G, Ye BH (2008). Constitutively activated STAT3 promotes cell proliferation and survival in the activated B-cell subtype of diffuse large B-cell lymphomas. Blood.

[31] Montes-Moreno S, Martinez N, Sanchez-Espiridion B, Diaz Uriarte R, Rodriguez ME, Saez A, Montalban C, Gomez G, Pisano DG, Garcia JF, Conde E, Gonzalez-Barca E, Lopez A, Mollejo M, Grande C, Martinez MA (2011). miRNA expression in diffuse large B-cell lymphoma treated with chemoimmunotherapy. Blood.

[32] Visco C, Li Y, Xu-Monette ZY, Miranda RN, Green TM, Tzankov A, Wen W, Liu W-M, Kahl BS, d'Amore ESG, Montes-Moreno S, Dybkaer K, Chiu A, Tam W, Orazi A, Zu Y (2012). Comprehensive gene expression profiling and immunohistochemical studies support application of immunophenotypic algorithm for molecular subtype classification in diffuse large B-cell lymphoma: a report from the International DLBCL Rituximab-CHOP Consortium Program Study. Leukemia.

[33] Hu S, Xu-Monette ZY, Tzankov A, Green T, Wu L, Balasubramanyam A, Liu WM, Visco C, Li Y, Miranda RN, Montes-Moreno S, Dybkaer K, Chiu A, Orazi A, Zu Y, Bhagat G (2013). MYC/BCL2 protein coexpression contributes to the inferior survival of activated B-cell subtype of diffuse large B-cell lymphoma and demonstrates high-risk gene expression signatures: a report from The International DLBCL Rituximab-CHOP Consortium Program. Blood.

[34] Lam LT, Wright G, Davis RE, Lenz G, Farinha P, Dang L, Chan JW, Rosenwald A, Gascoyne RD, Staudt LM (2008). Cooperative signaling through the signal transducer and activator of transcription 3 and nuclear factor-{kappa}B pathways in subtypes of diffuse large B-cell lymphoma. Blood.

[35] Visco C, Li Y, Xu-Monette ZY, Miranda RN, Green TM, Tzankov A, Wen W, Liu WM, Kahl BS, d'Amore ES, Montes-Moreno S, Dybkær K, Chiu A, Tam W, Orazi A, Zu Y (2012). Comprehensive gene expression profiling and immunohistochemical studies support application of immunophenotypic algorithm for molecular subtype classification in diffuse large B-cell lymphoma: A report from the International DLBCL Rituximab-CHOP consortium program study. Leukemia.

[36] Sartore-Bianchi A, Fieuws S, Veronese S, Moroni M, Personeni N, Frattini M, Torri V, Cappuzzo F, Vander Borght S, Martin V, Skokan M, Santoro A, Gambacorta M, Tejpar S, Varella-Garcia M, Siena S (2012). Standardisation of EGFR FISH in colorectal cancer: results of an international interlaboratory reproducibility ring study. Journal of clinical pathology.

[37] Ventura RA, Martin-Subero JI, Jones M, McParland J, Gesk S, Mason DY, Siebert R (2006). FISH analysis for the detection of lymphoma-associated chromosomal abnormalities in routine paraffin-embedded tissue. The Journal of molecular diagnostics: JMD.

[38] Iqbal J, Greiner TC, Patel K, Dave BJ, Smith L, Ji J, Wright G, Sanger WG, Pickering DL, Jain S, Horsman DE, Shen Y, Fu K, Weisenburger DD, Hans CP, Campo E (2007). Distinctive patterns of BCL6 molecular alterations and their functional consequences in different subgroups of diffuse large B-cell lymphoma. Leukemia.

[39] Tzankov A, Xu-Monette ZY, Gerhard M, Visco C, Dirnhofer S, Gisin N, Dybkaer K, Orazi A, Bhagat G, Richards KL, Hsi ED, Choi WW, van Krieken JH, Ponzoni M, Ferreri AJ, Ye Q (2013). Rearrangements of MYC gene facilitate risk stratification in diffuse large B-cell lymphoma patients treated with rituximab-CHOP. Modern pathology.

[40] Vose JM (2011). Intensified chemotherapy for diffuse large B-cell lymphomas. Lancet.

[41] Scott DW, Mottok A, Ennishi D, Wright GW, Farinha P, Ben-Neriah S, Kridel R, Barry GS, Hother C, Abrisqueta P, Boyle M, Meissner B, Telenius A, Savage KJ, Sehn LH, Slack GW (2015). Prognostic Significance of Diffuse Large B-Cell Lymphoma Cell of Origin Determined by Digital Gene Expression in Formalin-Fixed Paraffin-Embedded Tissue Biopsies. Journal of clinical oncology.

[42] Scott DW, Wright GW, Williams PM, Lih CJ, Walsh W, Jaffe ES, Rosenwald A, Campo E, Chan WC, Connors JM, Smeland EB, Mottok A, Braziel RM, Ott G, Delabie J, Tubbs RR (2014). Determining cell-of-origin subtypes of diffuse large B-cell lymphoma using gene expression in formalin-fixed paraffin-embedded tissue. Blood.

[43] Visco C, Tzankov A, Xu-Monette ZY, Miranda RN, Tai YC, Li Y, Liu WM, d'Amore ES, Montes-Moreno S, Dybkaer K, Chiu A, Orazi A, Zu Y, Bhagat G, Wang HY, Dunphy CH (2013). Patients with diffuse large B-cell lymphoma of germinal center origin with BCL2 translocations have poor outcome, irrespective of MYC status: a report from an International DLBCL rituximab-CHOP Consortium Program Study. Haematologica.

[44] Meyer PN, Fu K, Greiner TC, Smith LM, Delabie J, Gascoyne RD, Ott G, Rosenwald A, Braziel RM, Campo E, Vose JM, Lenz G, Staudt LM, Chan WC, Weisenburger DD (2011). Immunohistochemical methods for predicting cell of origin and survival in patients with diffuse large B-cell lymphoma treated with rituximab. Journal of clinical oncology.

[45] Choi WW, Weisenburger DD, Greiner TC, Piris MA, Banham AH, Delabie J, Braziel RM, Geng H, Iqbal J, Lenz G, Vose JM, Hans CP, Fu K, Smith LM, Li M, Liu Z (2009). A new immunostain algorithm classifies diffuse large B-cell lymphoma into molecular subtypes with high accuracy. Clin Cancer Res.

[46] Zhang LH, Kosek J, Wang M, Heise C, Schafer PH, Chopra R (2013). Lenalidomide efficacy in activated B-cell-like subtype diffuse large B-cell lymphoma is dependent upon IRF4 and cereblon expression. British journal of haematology.

[47] Dunleavy K, Pittaluga S, Czuczman MS, Dave SS, Wright G, Grant N, Shovlin M, Jaffe ES, Janik JE, Staudt LM, Wilson WH (2009). Differential efficacy of bortezomib plus chemotherapy within molecular subtypes of diffuse large B-cell lymphoma. Blood.

[48] Dasmahapatra G, Patel H, Dent P, Fisher RI, Friedberg J, Grant S (2013). The Bruton tyrosine kinase (BTK) inhibitor PCI-32765 synergistically increases proteasome inhibitor activity in diffuse large-B cell lymphoma (DLBCL) and mantle cell lymphoma (MCL) cells sensitive or resistant to bortezomib. British journal of haematology.

[49] Nowakowski GS, Czuczman MS (2015). ABC, GCB, and Double-Hit Diffuse Large B-Cell Lymphoma: Does Subtype Make a Difference in Therapy Selection?. American Society of Clinical Oncology educational book / ASCO American Society of Clinical Oncology Meeting.

[50] Delgado MD, Albajar M, Gomez-Casares MT, Batlle A, Leon J (2013). MYC oncogene in myeloid neoplasias. Clinical & translational oncology.

[51] Delmore JE, Issa GC, Lemieux ME, Rahl PB, Shi J, Jacobs HM, Kastritis E, Gilpatrick T, Paranal RM, Qi J, Chesi M, Schinzel AC, McKeown MR, Heffernan TP, Vakoc CR, Bergsagel PL (2011). BET bromodomain inhibition as a therapeutic strategy to target c-Myc. Cell.

[52] Ott CJ, Kopp N, Bird L, Paranal RM, Qi J, Bowman T, Rodig SJ, Kung AL, Bradner JE, Weinstock DM (2012). BET bromodomain inhibition targets both c-Myc and IL7R in high-risk acute lymphoblastic leukemia. Blood.

[53] Mertz JA, Conery AR, Bryant BM, Sandy P, Balasubramanian S, Mele DA, Bergeron L, Sims RJ (2011). Targeting MYC dependence in cancer by inhibiting BET bromodomains. Proceedings of the National Academy of Sciences of the United States of America.

[54] Gupta M, Maurer MJ, Wellik LE, Law ME, Han JJ, Ozsan N, Micallef IN, Dogan A, Witzig TE (2012). Expression of Myc, but not pSTAT3, is an adverse prognostic factor for diffuse large B-cell lymphoma treated with epratuzumab/R-CHOP. Blood.

[55] Paik JH, Nam SJ, Kim TM, Heo DS, Kim CW, Jeon YK (2014). Overexpression of sphingosine-1-phosphate receptor 1 and phospho-signal transducer and activator of transcription 3 is associated with poor prognosis in rituximab-treated diffuse large B-cell lymphomas. BMC cancer.

[56] Kiuchi N, Nakajima K, Ichiba M, Fukada T, Narimatsu M, Mizuno K, Hibi M, Hirano T (1999). STAT3 is required for the gp130-mediated full activation of the c-myc gene. The Journal of experimental medicine.

[57] Munoz J, Dhillon N, Janku F, Watowich SS, Hong DS (2014). STAT3 inhibitors: finding a home in lymphoma and leukemia. The oncologist.

[58] Ok CY, Chen J, Xu-Monette ZY, Tzankov A, Manyam GC, Li L, Visco C, Montes-Moreno S, Dybkaer K, Chiu A, Orazi A, Zu Y, Bhagat G, Richards KL, Hsi ED, Choi WW (2014). Clinical implications of phosphorylated STAT3 expression in De Novo diffuse large B-cell lymphoma. Clinical cancer research.

[59] Green TM, Young KH, Visco C, Xu-Monette ZY, Orazi A, Go RS, Nielsen O, Gadeberg OV, Mourits-Andersen T, Frederiksen M, Pedersen LM, Møller MB (2012). Immunohistochemical double-hit score is a strong predictor of outcome in patients with diffuse large B-cell lymphoma treated with rituximab plus cyclophosphamide, doxorubicin, vincristine, and prednisone. J Clin Oncol.

[60] Johnson NA, Slack GW, Savage KJ, Connors JM, Ben-Neriah S, Rogic S, Scott DW, Tan KL, Steidl C, Sehn LH, Chan WC, Iqbal J, Meyer PN, Lenz G, Wright G, Rimsza LM (2012). Concurrent expression of MYC and BCL2 in diffuse large B-cell lymphoma treated with rituximab plus cyclophosphamide, doxorubicin, vincristine, and prednisone. Journal of clinical oncology.

[61] Visco C, Tzankov A, Xu-Monette ZY, Miranda RN, Tai YC, Li Y, Liu WM, d'Amore ES, Montes-Moreno S, Dybkær K, Chiu A, Orazi A, Zu Y, Bhagat G, Wang HY, Dunphy CH (2013). Patients with diffuse large B-cell lymphoma of germinal center origin with BCL2 translocations have poor outcome, irrespective of MYC status: a report from an International DLBCL rituximab-CHOP Consortium Program Study. Haematologica.

[62] Horn H, Ziepert M, Wartenberg M, Staiger AM, Barth TF, Bernd HW, Feller AC, Klapper W, Stuhlmann-Laeisz C, Hummel M, Stein H, Lenze D, Hartmann S, Hansmann ML, Moller P, Cogliatti S (2015). Different biological risk factors in young poor-prognosis and elderly patients with diffuse large B-cell lymphoma. Leukemia.

[63] Lam LT, Zhang H, Chyla B (2012). Biomarkers of therapeutic response to BCL2 antagonists in cancer. Molecular diagnosis & therapy.

[64] Shustik J, Han G, Farinha P, Johnson NA, Ben Neriah S, Connors JM, Sehn LH, Horsman DE, Gascoyne RD, Steidl C (2010). Correlations between BCL6 rearrangement and outcome in patients with diffuse large B-cell lymphoma treated with CHOP or R-CHOP. Haematologica.

[65] Winter JN, Weller EA, Horning SJ, Krajewska M, Variakojis D, Habermann TM, Fisher RI, Kurtin PJ, Macon WR, Chhanabhai M, Felgar RE, Hsi ED, Medeiros LJ, Weick JK, Reed JC, Gascoyne RD (2006). Prognostic significance of Bcl-6 protein expression in DLBCL treated with CHOP or R-CHOP: a prospective correlative study. Blood.

[66] Malumbres R, Chen J, Tibshirani R, Johnson NA, Sehn LH, Natkunam Y, Briones J, Advani R, Connors JM, Byrne GE, Levy R, Gascoyne RD, Lossos IS (2008). Paraffin-based 6-gene model predicts outcome in diffuse large B-cell lymphoma patients treated with R-CHOP. Blood.

[67] Hatzi K, Melnick A (2014). Breaking bad in the germinal center: how deregulation of BCL6 contributes to lymphomagenesis. Trends in molecular medicine.

