# Supplementary material for: Stratifying diffuse large B-cell lymphoma patients treated with chemoimmunotherapy: GCB/non-GCB by immunohistochemistry is still a robust and feasible marker
Source: Oncotarget. 2016 Feb 19;7(14):18036–49. doi: 10.18632/oncotarget.7495 (PMC4951269; doi:10.18632/oncotarget.7495)
Supplement: Supplementary file 1 [file oncotarget-07-18036-s001.pdf]

Stratifying diffuse large B-cell lymphoma patients treated with chemoimmunotherapy: GCB/non-GCB by immunohistochemistry is still a robust and feasible marker

SUPPLEMENTARY DATA

MATERIALS AND METHODS

Immunohistochemistry and TMA construction

We used a tissue arrayer device (Beecher Instruments, Sun Prairie, WI) to construct tissue microarray (TMA) blocks, according to conventional protocols. Standard tissue sections were also analyzed in some cases. Immunohistochemical staining was performed as follows: 2-4-µm-thick paraffin-embedded TMAs were cut onto Dako slides (Dako, Agilent technologies, California, USA), and subsequently dewaxed, rehydrated and subjected to antigen retrieval by heating in 50 mM Tris (Trizma base)-1 mMEDTA (ethylenediaminetetraacetic acid) (Sigma Chemicals, St. Louis, USA,) (pH 8) or citrate 10 mM pH 6.5 in a pressure cooker for 2 min. The slides were cooled and treated with peroxidase-blocking solution (Dako) for 5 min. Sections were then immunostained with antibodies against Gcet1, MUM1, CD10, BCL6 FOXP1, C-MYC, BCL2 and pSTAT3, counterstained with hematoxylin and mounted. Incubations, either omitting the specific antibody or containing unrelated antibodies, were used as a negative control of the technique.

The antibodies used were CD10 (clone 56C6, RTU, DAKO), bcl6 (clone PG-B6p, RTU, DAKO), MUM-1/IRF4 (clone MUM1P, RTU, DAKO), Gcet1 (clone RAM, dilution 1:4, CNIO, Madrid, Spain) and FOXP1 (clone JC12, dilution 1:100, CNIO), C-MYC (EP121 Epitomics), BCL2 (clone 124 DAKO), pSTAT3 (clone EP2147Y, Chemicon).

INTERPHASE FISH ANALYSIS

FISH analyses were performed on 3-µm TMA tissue sections applying 10 µl of commercial probes against *MYC* (LSI MYC Dual Color, Break Apart,01N63-020, Abbott Molecular, Abbott Park, IL, USA), *BCL2* (LSI BCL2 (18q21) Dual Color, Break Apart, 05N51-020, Abbott Molecular), *BCL6* (LSI BCL6 Dual Color, Break Apart, 01N23-020, Abbott Molecular) or *IRF4* (RF™ POSEIDON™ IRF4 / DUSP22 (6p25) Break Apart Probe, KBI-10613, KREATECH Diagnostics, Amsterdam, The Netherlands).

REFERENCES

1. García JF, Maestre L, Lucas E, et al: Genetic immunization: a new monoclonal antibody for the detection of BCL-6 protein in paraffin sections. J Histochem Cytochem 54:31-8, 2006.

2. Montes-Moreno S, Roncador G, Maestre L, et al: Gcet1 (centerin), a highly restricted marker for a subset of germinal center-derived lymphomas. Blood 111:351-358, 2008.

Supplementary Table S1: Main clinical and biological characteristics

	All DLBCL (n=297)
**Age, years**	
> 60	166 of 278 (60)
≤ 60	112 of 278 (40)
**Ann Arbor stage**	
I-II	113 of 269 (42)
III-IV	156 of 269 (58)
**LDH**	
Low	101 of 243 (42)
> upper limit of normal	142 of 243 (58)
**Extranodal sites**	
≤ 1	175 of 238 (74)
≥ 2	63 of 238 (26)
**ECOG †**	
PS ≤ 1	166 of 239 (69)
PS > 1	73 of 239 (31)
**IPI score ‡**	
Low (0-2)	151 of 277 (54.5)
High (3-5)	126 of 277 (45.5)
**Cell of origin subtype**	
Hans Classification	
Non-GCB	157 of 272 (58)
GCB	115 of 272 (42)
Choi classification	
NON-GCB	154 of 272 (57)
GCB	118 of 272 (43)
Visco-Young classification	
NON-GCB	170 of 282 (60)
GCB	112 of 282 (40)
**MYC**	
MYC translocation	15 of 206 (7)
MYC gain	37 of 191 (19)
Positive MYC protein expression	62 of 140 (44)
**BCL2**	
BCL2 translocation	36 of 187 (19)
BCL2 gain	25 of 181 (14)
Positive BCL2 protein expression	83 of 138 (60)
**BCL6**	
BCL6 translocation	51 of 171 (30)
BCL6 gain	30 of 180 (17)
Positive BCL6 protein expression	200 of 281 (71)
**pSTAT3** protein expression	50 of 156 (32)
**IRF4**	
IRF4 translocation	3 of 76 (4)
IRF4 gain	4 of 41 (10)
**IHC** MYC+/BCL2+	42 of 126 (33)
**FISH** *MYC+/BCL2 + MYC+/BCL6+*	5 of 126 (4)

Abbreviations: DLBCL, diffuse large B-cell lymphoma; ECOG PS, Eastern Cooperative Oncology Group performance status; GCB, germinal center B-cell-like; IPI, International Prognostic Index; LDH, lactate dehydrogenase; †ECOG PS ranges from 0 to 4, where a higher score indicates greater impairment. ‡IPI score ranges from 0 to 5, with 0 indicating the absence of prognostic factors and 5 indicating the presence of all prognostic factors; IHC: Immunohistochemical.

Supplementary Table S2: International prognostic index (IPI) and *MYC*, *BCL2*, *BCL6* and *IRF4* abnormalities according to the cell of origin classification (GCB vs. non-GCB)

	Choi classification (n=275)					Visco-Young classification (n=282)					Hans classification (n=281)				
	GCB(n=120)		Non GCB(n=155)			GCB(n=112)		NON-GCB(n=170)			GCB (n=121)		Non GCB(n=160)		
**Cohort**	No	%	No	%	*p*	No	%	No	%	*p*	No	%	No	%	*p*
Age, years															
> 60	66 of 114	58	92 of 146	63	0.402	62 of 106	59	98 of 159	62	0.608	65 of 114	57	95 of 150	63	0.298
≤ 60	48 of 114	42	54 of 146	37		44 of 106	41	61 of 159	38		49 of 114	43	55 of 150	37	
Ann Arbor stage III-IV	52 of 110	47	97 of 144	67	**0.001**	47 of 102	46	104 of 156	67	**0.001**	50 of 108	46	102 of 147	69	<**0.001**
LDH > upper limit of normal	53 of 99	53	80 of 128	63	0.174	45 of 91	50	90 of 141	64	**0.030**	49 of 98	50	86 of 132	65	**0.021**
Extranodal sites ≤ 2	25 of 98	25	33 of 125	26	0.881	24 of 91	26	35 of 137	26	.0889	21 of 97	22	38 of 128	30	0.175
ECOG PS > 1†	23 of 97	24	43 of 126	34	0.091	17 of 90	19	50 of 138	36	**0.005**	20 of 97	21	48 of 129	37	**0.007**
IPI score ‡ High (3-5)	43 of 116	37	77 of 146	53	**0.011**	39 of 107	36	83 of 159	52	**0.011**	38 of 112	34	84 of 151	56	**<0.001**

Abbreviations: GCB: germinal center B-cell-like; DLBCL, diffuse large B-cell lymphoma; ECOG PS, Eastern Cooperative Oncology Group performance status; IPI, International Prognostic Index; LDH: lactate dehydrogenase; DHL double-hit lymphoma; †ECOG PS ranges from 0 to 4, where a higher score indicates greater impairment. ‡IPI score ranges from 0 to 5, with 0 indicating the absence of prognostic factors and 5 indicating the presence of all prognostic factors.

Supplementary Table S3: Clinical features according to the presence of MYC and BCL6 protein expression

	MYC protein expression (n=62/140)			BCL6 protein expression (n=200/281)		
**Patients, No. (%)**	Negative (n=78)	Positive (n=62)	P	Negative (n=81)	Positive (n=200)	P
**Age, years**						
> 60	40 (58)	38 (72)	0.118	42 (57)	119 (63)	0.379
≤ 60	29 (42)	15 (28)		32 (43)	71 (37)	
**Ann Arbor stage**						
I-II	27 (40)	15 (29)	0.245	23 (31)	80 (44)	**0.053**
III-IV	41 (60)	36 (71)		51 (69)	101 (56)	
**LDH**						
Low	34 (51)	14 (27)	**0.011**	21 (32)	73 (44)	0.076
> upper limit of normal	33 (49)	37 (72)		45 (68)	91 (56)	
**Extranodal sites**						
≤ 1	45 (67)	36 (69)	0.810	45 (69)	121 (76)	0.323
≥ 2	21 (33)	16 (31)		20 (31)	39 (24)	
**ECOG †**						
PS ≤ 1	43 (63)	29 (56)	0.408	36 (54)	120 (75)	**0.002**
PS > 1	25 (37)	23 (43)		30 (46)	40 (25)	
**IPI score ‡I**						
Low (0-2)	38 (56)	19 (36)	**0.029**	35 (48)	105 (55)	0.287
High (3-5)	30 (44)	34 (64)		38 (52)	85 (45)	
**MYC**						
r*MYC*	3 (5)	5 (10)	0.334	4 (7)	10 (7)	0.926
g*MYC*	11 (20)	5 (11)	0.196	6 (12)	31 (23)	0.092
**BCL2**						
r*BCL2*	13 (30)	8 (18)	0.211	1 (2)	34 (24)	**0.001**
g*BCL2*	7 (16)	4 (9)	0.375	9 (21)	16 (12)	0.115
**BCL6**						
r*BCL6*	15(32)	15 (31)	0.891	12 (28)	39 (31.5)	0.664
g*BCL6*	5(12)	4(8)	0.596	2 (5)	28 (21)	**0.018**
**pSTAT3** protein expression	15(21)	26 (44)	**0.006**	15 (30)	34 (35)	0.566
**IRF4**						
r*IRF4*	2 (5)	0	0.247	0	3 (6)	0.198
g*IRF4*	4 (19)	0	0.122	3 (18)	1 (4)	0.152

Abbreviations: IPI, International Prognostic Index; LDH, lactate dehydrogenase; r*MYC*: rearrangement of *MYC*; g*MYC*: gain of *MYC* locus; r*BCL2*: rearrangement of *BCL2*; g*BCL2*: gain of *BCL2* locus; r*BCL6* rearrangement of *BCL6;* g*BCL6*: gain of *BCL6* locus †ECOG PS ranges from 0 to 4, where a higher score indicates greater impairment. ‡ IPI score ranges from 0 to 5, with 0 indicating the absence of prognostic factors and 5 indicating the presence of all prognostic factors.

Supplementary Table S4: MYC, BCL2, BCL6, pSTAT3 pattern expression according to the cell of origin classification

	Choi classification (n=275)					Visco-Young classification (n=282)					Hans classification (n=281)				
	GBC (n=120)		Non-GCB (n=155)			GCB (n=112)		Non-GCB (n=170)			GBC (n=121)		Non-GBC (n=160)		
**Cohort**	No	%	No	%	p	No	%	No	%	p	No	%	No	%	p
MYC protein expression	18 of 51	35	39 of 77	51	0.087	20 of 52	39	41 of 82	50	0.191	16 of 44	36	43 of 89	48	0.192
BCL2 protein expression	27 of 52	52	53 of 76	70	**0.041**	30 of 52	58	51 of 81	63	0.543	23 of 45	51	57 of 86	66	0.091
BCL6 protein expression	104 of 116	90	88 of 154	57	**<0.001**	109 of 119	92	90 of 159	57	**0.000**	100 of 110	91	97 of 166	58	**<0.001**
pSTAT3 protein expression	14 of 54	26	32 of 90	35	0.230	11 of 53	21	39 of 95	41	**0.012**	11 of 45	24	37 of 103	36	0.170
MYC and BCL2 protein expression	8 of 46	17	33 of 72	46	**0.002**	7 of 40	17	34 of 81	42	**0.007**	10 of 47	21	32 of 76	42	**0.018**

Abbreviations: DLBCL, diffuse large B-cell lymphoma; ECOG PS, Eastern Cooperative Oncology Group performance status; GCB, germinal center B-cell-like.

Supplementary Table S5: Clinical characteristics of immunohistochemical double-positive cases and pSTAT3 protein expression

	IHC Double-positive		pSTAT3	
Patients, No. (%)		P		P
**Age, years**				
> 60	29 (74)		28 (60)	
≤ 60	10 (26)	0.088	19 (40)	0.690
**Ann Arbor stage**				
I-II	10 (26)		9 (20)	
III-IV	29 (74)	0.111	37 (80)	**0.002**
**LDH**				
Low	7 (18)		17 (38)	
> upper limit of normal	32 (82)	**<0.001**	28 (62)	0.709
**Extranodal sites**				
≤ 1	25 (64)	0.599	34 (74)	0.633
≥ 2	14 (36)		12 (26)	
**ECOG**				
PS ≤1	22 (56)	0.531	26 (56)	0.108
PS >1	17 (44)		20 (44)	
**IPI score**				
Low (0-2)	13 (32)	**0.005**	19 (40)	**0.041**
High (3-5)	28 (68)		28 (60)	

Abbreviations: ECOG PS, Eastern Cooperative Oncology Group performance status; GCB, germinal center B-cell-like; GEP, gene expression profiling; IPI, International Prognostic Index; LDH, lactate dehydrogenase; †ECOG PS ranges from 0 to 4, where a higher score indicates greater impairment. ‡IPI score ranges from 0 to 5, with 0 indicating the absence of prognostic factors and 5 indicating the presence of all prognostic factors.

Supplementary Table S6: Gene abnormalities of immunohistochemical double-positive cases

	IHC Double-positive	
**Patients, No. (%)**		P
**Cell of origin subtype**		
Hans classification		
Non-GBC	33 (76)	**0.018**
GCB	10 (24)	
Choi classification		
Non-GCB	33 (81)	**0.002**
GCB	8 (19)	
Visco-Young classification		
Non-GCB	34 (83)	**0.007**
GCB	7 (17)	
MYC		
MYC translocation	5 (15)	**0.030**
MYC gains	3 (10)	0.232
Positive MYC protein expression	-	-
BCL2		
BCL2 translocation	8 (24)	0.938
BCL2 gains	3 (10)	0.522
Positive BCL2 protein expression	-	
BCL6		
BCL6 translocation	11 (32)	0.745
BCL6 gains	4 (12)	0.724
Positive BCL6 protein expression	29 (69)	0.647
**pSTAT3** protein expression	16 (39)	0.114
**IRF4**		
IRF4 translocation	0	0.439
IRF4 gains	0	0.139

Abbreviations: DLBCL, diffuse large B-cell lymphoma; GCB, germinal center B-cell-like.

Supplementary Table S7: Clinical features with respect to the presence of *MYC*, *BCL2* and *BCL6* gains

	***MYC*(n=37/191) Gained**		***BCL2*(n=25/181) Gained**		***BCL6*(n=30/180) Gained**	
**Patients, No. (%)**		P		P		P
**Age, years**						
> 60	26 (74)	0.167	3 (59)	0.672	18 (60)	0.862
≤ 60	9 (26)		9 (41)		12 (40)	
**Ann Arbor stage**						
I-II	16 (47)	0.254	9 (43)	546	12 (44)	0.740
III-IV	18 (53)		12 (57)		15 (56)	
**LDH**						
Low	18 (60)	**0.032**	9 (50)	0.501	8 (40)	0.576
> upper limit of normal	12 (40)		9 (59)		12 (60)	
**Extranodal sites**						
≤ 1	22 (73)	0.484	13 (72)	0.890	11 (61)	0.343
≥ 2	8 (27)		5 (28)		7 (39)	
**ECOG †**						
PS ≤ 1	23 (77)	0.330	13 (72)	0.578	1 (61)	0.482
PS > 1	7 (23)		5 (28)		7 (39)	
**IPI score ‡**						
Low (0-2)	19 (54)	0.650	13 (59)	0.303	17 (57)	0.812
High (3-5)	16 (46)		9 (41)		13 (43)	

Abbreviations: DLBCL, diffuse large B-cell lymphoma; ECOG PS, Eastern Cooperative Oncology Group performance status; GCB, germinal center B-cell-like; IPI, International Prognostic Index; LDH, lactate dehydrogenase; †ECOG PS ranges from 0 to 4, where higher score indicates greater degree of impairment. ‡IPI score ranges from 0 to 5, with 0 indicating absence of prognostic factors and 5 indicating presence of all prognostic factors.

Supplementary Table S8: Clinical and biological characteristics of double-hit cases

	FISH double-hit *BCL2*+/*MYC*+ (n=5/126)
**Age, years**	
> 60	4 (80)
≤ 60	1 (20)
**Ann Arbor stage**	
I-II	2 (40)
III-IV	3 (60)
**LDH**	
Low	1 (20)
> upper limit of normal	4 (80)
**Extranodal sites**	
≤ 1	3 (60)
≥ 2	2 (40)
**ECOG †**	
PS ≤ 1	3 (60)
PS > 1	2 (40)
**IPI score ‡I**	
Low (0,1,2)	3 (60)
High (3, 4 or 5)	2 (40)
**Cell of origin subtype**	
(Hans classification)	
Non-GBC	1 (20)
GBC	4 (80)
(Choi classification)	
NON-GCB	1 (20)
GBC	4 (80)
(Visco-Young classification)	
NON-GCB	1 (20)
GCB	4 (80)
**MYC**	
MYC translocation	5 (100)
MYC gained	1 (20)
Positive MYC protein expression	4 (100)
**BCL2**	
BCL2 translocation	4 (100)
BCL2 gains	0
Positive BCL2 protein expression	4 (100)
**BCL6**	
BCL6 translocation	1 (20)
BCL6 gains	0
Positive BCL6 protein expression	4 (80)
**pSTAT3 protein expression**	0
**IRF4**	
IRF4 translocation	0
IRF4 gained	0

Abbreviations: DLBCL, diffuse large B-cell lymphoma; ECOG PS, Eastern Cooperative Oncology Group performance status; GCB, germinal center B-cell-like; IPI, International Prognostic Index; LDH, lactate dehydrogenase; †ECOG PS ranges from 0 to 4, where higher score indicates greater degree of impairment. ‡IPI score ranges from 0 to 5, with 0 indicating absence of prognostic factors and 5 indicating presence of all prognostic factors; IHC: Immunohistochemical.

Supplementary Table S9: Progression free survival in the GCB group, with respect to immunohistochemical double-positive cases and abnormalities of *MYC*, *BCL2* and *BCL6*

	GCB-Hans		GCB-Choi		GCB-Visco	
	PFS		PFS		PFS	
	RR 95%CI	p	RR 95%CI	p	RR 95%CI	p
**MYC**						
r*MYC*	3.5 (1.09-11.25)	**0.035**	3.2 (1.03-10.28)	**0.044**	4.2 (1.2-14.5)	**0.021**
g*MYC*	0.5 (0.15-1.9)	0.346	0.5 (0.158-1.94)	0.355	0.89 (0.237-3.372)	0.869
MYC protein expression	1.5 (0.59-4)	0.376	1.1 (0.4-2.867)	0.811	0.765 (0.230-2.548)	0.662
**BCL2**						
r*BCL2*	1.9 (0.75-4.8)	0.172	1.7 (0.69-4.2)	0.246	2.01 (0.728-5.55)	0.178
g*BCL2*	0.8 (0.1-6.27)	0.852	0.7 (0.098-5.5)	0.770	0.9 (0.1-6.9)	0.922
BCL2 protein expression	1.4 (0.47-4.1)	0.544	1.5 (0.5-4.3)	0.422	2.0 (0.5-7.8)	0.309
**BCL6**						
r*BCL6*	1.2 (0.3-4.4)	0.752	.3 (0.370-4.761)	0.664	0.5 (0.06-3.9)	0.512
g*BCL6*	0.03 (0.0-4.3)	0.168	0.031 (0.0-3.761)	0.156	0.036 (0-23)	0.315
BCL6 protein expression	1.6 (0.3-6.9)	0.522	1.3 (0.38-4.47)	0.673	1.175 (0.269-5.137)	0.830
**MYC and BCL2** protein expression	2.5 (0.8-7.8)	0.100	2.3 (0.7-7.8)	0.153	4 (1-15)	**0.041**

Abbreviations: GCB: germinal center B-cell-like; DLBCL, diffuse large B-cell lymphoma; r*MYC*: translocation of *MYC*; g*MYC*: gain of *MYC* locus; r*BCL2*: translocation of *BCL2*; g*BCL2*: gain of *BCL2* locus; r*BCL6* translocation of *BCL6*; g*BCL6*: gain of *BCL6* locus.

Supplementary Table S10: Overall survival in the GCB group, with respect to immunohistochemical double-positive cases and abnormalities of *MYC*, *BCL2* and *BCL6*

	GCB-Hans		GCB-Choi		GCB-Visco	
	OS		OS		OS	
	RR 95%CI	p	RR 95%CI	p	RR 95%CI	p
MYC						
rMYC	2.5 (0.709-8.9)	0.153	2.2 (0.65-7.9)	0.198	2.9 (0.8-10.8)	0.098
g*MYC*	0.6 (0.178-2.2)	0.479	0.59 (0.17-2.05)	0.410	0.8 (0.2-3.1)	0.793
MYC protein expression	2.2 (0.69-7.4)	0.177	1.06 (0.3-3.5)	0.924	0.76 (0.1-4.19)	0.754
BCL2						
r*BCL2*	0.629 (0.2-1.98)	0.429	0.5 (0.167-1.5)	0.233	0.469 (0.13-1.68)	0.246
gBCL2	1.03 (0.133-8.0)	0.974	0.829 (0.1-6.3)	0.856	1.0 (0.132-7.9)	0.984
BCL2 protein expression	0.7 (0.2-2.7)	0.686	0.7 (0.19-2.5)	0.586	0.578 (0.096-3.46)	0.549
BCL6						
r*BCL6*	1.6 (0.5-5.28)	0.376	1.79 (0.57-5.6)	0.317	1.05 (0.2-4.8)	0.947
g*BCL6*	0.48 (0.109-2.1)	0.337	0.428 (0.09-1.8)	0.257	0.79 (0.175-3.56)	0.760
BCL6 protein expression	1.03 (0.236-4.57)	0.960	0.657 (0.08-5.3)	0.695	0.57 (0.154-2.1)	0.401
MYC and BCL2 protein expression	1.8 (0.46-7.5)	0.378	0.6 (0.08-5.3)	0.695	1.45 (0.150-14.1)	0.745

Abbreviations: GCB: germinal center B-cell-like; DLBCL, diffuse large B-cell lymphoma; *rMYC*: translocation of *MYC*; *gMYC*: gain of *MYC* locus; *rBCL2*: translocation of *BCL2*; *gBCL2*: gain of *BCL2* locus; *rBCL6* translocation of *BCL6*; *gBCL6*: gain of *BCL6* locus.
